# How University Students Evaluate the Use of Laboratory Animals: The Role of Species and Individual Differences

**DOI:** 10.3390/ani16071005

**Published:** 2026-03-25

**Authors:** Leire Ruiz-Sancho, Oihane Saez-Atxukarro, Ainara Gomez-Gastiasoro, Garikoitz Azkona

**Affiliations:** 1 Animal Facility Service (SGIker), Euskal Herriko Unibertsitatea (UPV/EHU), 48940 Leioa, Spain; leire.ruizs@ehu.eus; 2 Department of Health Sciences, Public University of Navarre, 31009 Pamplona, Spain; oihane.saez@unavarra.es; 3 Department of Basic Psychological Processes and Their Development, Euskal Herriko Unibertsitatea (UPV/EHU), 20018 San Sebastian, Spain; ainara.gomez@ehu.eus

**Keywords:** laboratory animal, biomedical research, anthropomorphism, empathy, personality

## Abstract

The use of animals in biomedical research and university teaching remains socially sensitive, shaped by ethical concerns, regulations, and public expectations. While animal models are still essential for basic and translational research, society increasingly demands transparency, strong welfare protections, and the development of alternative methods. This study investigated Spanish university students’ attitudes toward the use of animals in basic research, translational research, and educational settings, and examined their acceptance of different species for addressing human health problems. It also assessed how sociodemographic variables, personality traits, empathy, and anthropomorphism relate to these views. The sample included 653 students, predominantly women, heterosexual, and urban residents. Most participants supported the use of animals in research, whereas opinions regarding teaching uses were more divided. Attitudes toward the use of laboratory animals consistently differed by gender and field of study, with men and students in science-related disciplines showing higher acceptance. Of the psychological variables assessed, only anthropomorphism showed moderate negative correlations with support for the use of laboratory animals across all contexts. Species strongly influenced attitudes: companion animals generated the most opposition, primates and livestock elicited mixed responses, and rodents, invertebrates, and aquatic species received the highest support. Overall, students generally accept animal use but vary substantially by species, gender, and academic background.

## Simple Summary

This study explored how Spanish university students think about using animals in research and teaching. Most students supported animal use in scientific research, although opinions about using animals in university teaching were more mixed. Attitudes differed by gender and study field: men participants and students studying science-related subjects were more supportive. Students were less willing to use companion animals, showed mixed views on primates and livestock, and were most accepting of rodents, invertebrates, and aquatic species. Of the psychological variables assessed, only anthropomorphism showed moderate negative correlations with support for laboratory animal use. Overall, participants in the study generally accept animal use in biomedical research, but their support varies by species, gender, and academic background.

## 1. Introduction

Throughout history, philosophers have often distinguished humans from other animals. Aristotle placed humans at the top of a natural hierarchy, while Descartes viewed animals as mere machines without sentience. Kant acknowledged animal suffering but denied them moral status due to their lack of autonomy [1]. Later, utilitarianism introduced a consequentialist approach, arguing that actions are justified when they produce more benefits than harm, such as animal research being acceptable only if societal gains outweigh animal suffering [2]. The 19th century witnessed the emergence of animal protection movements [3]. By the mid-20th century, ethical debates surrounding speciesism and animal rights had gained prominence [4,5,6]. In this context, international ethical guidelines for research involving human subjects, such as those derived from the Declaration of Helsinki and the Nuremberg Code, establish that medical research on humans must be grounded in prior evidence, including results obtained from animal experimentation [7]. Recent perspectives advocate for frameworks grounded in justice and compassion, challenging the instrumental use of animals for human benefit [8]. In this regard, three broad stances have been identified among society: (1) human beings have a moral importance that other animals lack; (2) a sliding moral scale exists in which humans are at the top; and (3) human beings and animals are moral equals [9].

Although alternative modelling approaches have advanced significantly, animal models continue to play a crucial role in biomedical basic and translational research [10,11]. Current ethical standards require that clinical trials be supported by prior animal research [7]. The use of animals in science follows the principles of replacement, reduction, and refinement (3Rs) [12], as established by the European Directive 2010/63/EU, which Member States implemented in 2013 [13]. That same year, the EU’s full ban on cosmetic animal testing came into force [14]. European legislation sets the long-term goal of fully replacing procedures involving live animals whenever scientifically feasible, a shift strongly supported by citizens: a recent poll in eight EU countries found that 77% favour a transition to non-animal science and 75% believe the EU should lead global innovation in this area [15]. However, this objective remains distant. For example, the Netherlands’ 2016 plan to eliminate animal testing for regulatory safety by 2025 has not been achieved and now requires a longer-term strategy [16]. Meanwhile, the scientific community continues working to implement the 3Rs while calling for greater resources and interdisciplinary collaboration to advance alternatives as quickly as possible [17,18,19].

A wide range of biological and sociocultural factors shapes public attitudes toward the use of laboratory animals in biomedical research. Individual characteristics play a key role in influencing these views. Women, younger people, urban residents, and individuals with lower educational levels or without a scientific background tend to express stronger opposition to animal experimentation [20,21,22,23,24]. Opinions are also influenced by prior or current experiences with animals [25,26,27]. Furthermore, the degree of concern varies between countries and depends on the specific species involved.

Spain is among the European countries most accepting of biomedical animal research, according to studies conducted over the past two decades. Early research among psychology students showed that 66% supported animal experimentation, especially for medical purposes, and considered it necessary for scientific progress [23]. At the societal level, more recent surveys reveal similar trends: 73.1% of Spaniards approved the use of animals for research [22]. However, another study indicates that 76% of Spaniards report being very concerned about the use of animals in scientific research, testing, and education, and 82% believe that the EU should lead the transition toward innovation that does not rely on animal use [15]. In a European comparison, Spanish citizens expressed greater concern for the welfare of laboratory animals than respondents in Romania, Lithuania, Poland, or Sweden [28]. Similarly, the majority of individuals working with laboratory animals in Spain are in favour of their use and highly aware of, and sensitive to, both animal welfare and their own wellbeing [29,30,31]. However, many avoid discussing their work outside their immediate social circle [26,31]. Overall, these findings show that while animal use in biomedical research is broadly accepted in Spain, society expects strong welfare protections and the adoption of alternatives whenever possible. They also confirm that animal experimentation remains a sensitive and contested issue.

When considering the species used, support for certain animals has declined in Spain. The Special Eurobarometer (2010) indicated that 75% of Spaniards considered the use of mice acceptable, and 65% approved the use of dogs and monkeys for health-related studies [32]. Since then, support for using dogs and monkeys has dropped significantly. The latest surveys confirm that attitudes are strongly influenced by the species involved and the perceived moral status: mice remain the most accepted, pigs occupy an intermediate position, while dogs and non-human primates elicit the greatest opposition [22,26]. It is important to note that in Spain, dogs are regarded as beloved companion animals, forming strong emotional bonds [33], whereas rodents are often perceived as pests, and farm animals as profit [34].

Spain has progressively adopted stricter ethical standards, greater transparency, and a stronger push for alternatives while still recognizing the importance of biomedical research for human health [35]. In this context, the study investigates Spanish university students’ attitudes toward using animals in basic and translational research and in university teaching, including their preferences regarding the species involved. Because students represent future scientists, policymakers, educators, and informed citizens, their views offer an early indicator of shifting societal expectations about animal use in science. The study also examines how sociodemographic factors and psychological variables, particularly personality traits, empathy, and anthropomorphism, shape these attitudes, providing a clearer picture of the factors that influence support for or opposition to animal use in scientific and educational settings.

## 2. Materials and Methods

Participants were recruited online between 15 September and 23 December 2025, through the official student email lists of the Public University of Navarre (UPNA) and the University of the Basque Country (UPV/EHU), complemented by snowball sampling to reach students outside institutional mailing lists. Only individuals enrolled at Spanish universities were eligible. The introductory letter explained that data would be used solely for research and that anonymity was guaranteed. Participation was voluntary, informed consent was obtained, and the 10 min survey was completed via Microsoft Forms. The study followed the Declaration of Helsinki and received approval from the Ethics Committee for Human-Related Research (CEISH) of the University of the Basque Country (UPV/EHU); PI_2025_020.

The study used several validated instruments to assess attitudes toward animal use, psychological traits, and sociodemographic characteristics. Specifically, we employed the following measures:

Sociodemographic Variables: Participants were asked about their gender, age, sexual orientation, area of residence (urban or rural), and field of study (classified as belonging to Humanities or Sciences). Humanities included fields such as philosophy, ethics, history, and cultural studies, while Sciences comprised disciplines such as biology, medicine, or pharmacy.

Personality: The Spanish version of the Ten Item Personality Inventory (TIPI) was administered [36]. This brief instrument measures the five major personality dimensions, agreeableness (tendency toward empathy and cooperation), conscientiousness (organization and responsibility), emotional stability (ability to remain calm and resilient under stress), extraversion (sociability and assertiveness), and openness to experience (curiosity and preference for novelty), through two items per dimension (one positively and one negatively worded). Responses are rated on a 7-point Likert scale ranging from 1 (totally disagree) to 7 (totally agree). Scores for each dimension were computed by averaging the corresponding items after reverse-coding when appropriate, with higher scores indicating a greater presence of the trait.

Empathy: The Spanish short version of the Toronto Empathy Questionnaire (TEQ), a reduced scale of 11 items designed to assess empathy as a unidimensional construct, was used in the study [37]. The scale consists of 11 items assessing empathy as a unidimensional construct. Items 7, 11, and 15 of the original long version correspond to the reverse-coded items retained in the short Spanish adaptation. These items were reverse-coded prior to computing the total score, following the validated scoring procedure of the Spanish short TEQ. Internal consistency in the present sample was excellent (α = 0.824).

Anthropomorphism: This instrument was adapted to assess cognitive and emotional attributions toward animals [34]. The anthropomorphism scale was adapted from the original version, which referred specifically to companion animals (e.g., “I think my pet…”). Because the aim of the present study was to assess general attributions toward animals rather than attitudes toward pets, the referent was modified to “I think that animals…”. Only the introductory phrase was changed; the content of the cognitive and emotional attribution items remained identical to the original instrument. This adaptation allowed the scale to be applied to a broader range of species without altering the underlying construct. Reliability remained excellent (α = 0.875), indicating that the modification did not compromise internal consistency.

Attitudes Toward the Use of Animals: Participants were asked to indicate their level of agreement with the following statements: (a) Basic biomedical research (understanding essential biological mechanisms without immediate clinical purposes, but with potential for future health advances); (b) Translational or applied biomedical research (transforming basic knowledge into practical medical solutions, accelerating the transition from laboratory to patient); (c) University teaching. Responses were recorded on a five-point ordinal scale: ‘totally agree,’ ‘agree,’ ‘neither agree nor disagree,’ ‘disagree,’ and ‘totally disagree.’ For statistical analysis, these categories were coded numerically from −2 to 2, with 0 representing the neutral option (‘neither agree nor disagree’). This transformation enabled correlation analyses and other quantitative procedures [38]. Finally, using the same response options, participants were asked to respond to the following statement: “Scientists should be allowed to do research on animals such as (animal species) if it produces new information about human health problems.” [26,32]. Participants were presented with a predefined list of animal species and asked to rate it. This list was designed to cover a wide range of species commonly considered in biomedical research and education, allowing us to examine differences in attitudes across species categories.

All analyses were conducted in Jamovi (version 2.3.28.0, Sidney, Australia). Descriptive statistics summarized the sample using frequencies and means with standard deviations (SD). Because all variables failed the Shapiro–Wilk normality test, non-parametric tests were applied: Mann–Whitney U for two-category variables and Kruskal–Wallis tests for variables with more than two categories. Effect sizes were calculated using rank biserial correlation (rrb; <0.3 small, 0.3–0.5 moderate, >0.5 large) and the Squared Epsilon coefficient (ε^2^; 0.01–0.06 small, 0.06–0.14 moderate, ≥0.14 large). Chi-square tests assessed associations between opinions and demographic variables, with Cramer’s V as the effect size (≤0.2 weak, 0.2–0.4 moderate, >0.4 strong). Spearman’s rho was used to examine correlations between parameters (≤0.29 small, 0.30–0.49 moderate, ≥0.50 large). Responses marked “Prefer not to answer” and categories with fewer than 10 participants were excluded. Differences were considered statistically significant at *p* < 0.05 and highly significant at *p* < 0.01. Small effect sizes are reported in the Results but were not considered in the interpretation.

## 3. Results

### 3.1. Participant Information

A total of 711 individuals began the survey, but 58 (8.11%) did not provide consent, leaving a final sample of 653 participants. Most identified as women (69.9%), were heterosexual (67.6%), and lived in urban areas (79.2%). The mean age was 20.3 years (SD = 2.41). Most students were enrolled in science-related degrees (68.6%) and were in their first year (41.5%). Demographic characteristics appear in Table 1.

A highly significant gender difference was found for empathy, with women scoring higher than men (35.5 ± 4.11 vs. 31.9 ± 5.46; U = 23,672, *p* < 0.001, rrb = 0.418). No other psychological variables showed significant gender differences. No significant differences were observed for the remaining psychological variables. Psychological variable scores are described in Table 2.

### 3.2. Participants’ Opinions on Animal Use in Research and Teaching

**Figure 1:**
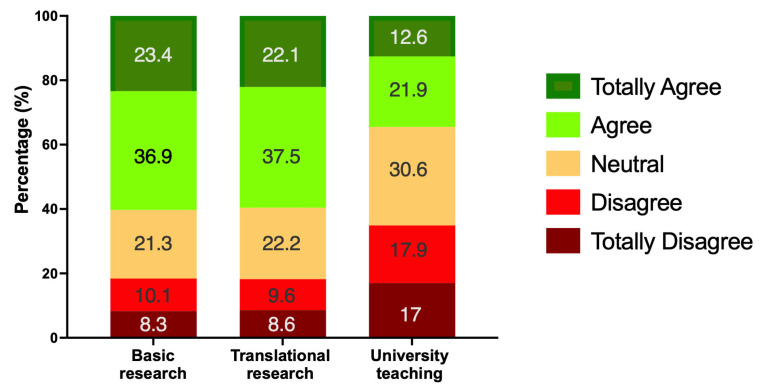
Percentage of responses to the question “I consider the use of animals ethical in…” across three contexts: Basic Research, Translational Research, and University Teaching. Reponses were recorded on a scale from “totally disagree” to “totally agree”. Bars represent the proportion of participants selecting each category.

When asked about the importance of using animals in different settings, responses varied across basic research, translational research, and university teaching (Figure 1; Supplementary Table S1). For basic research, most participants expressed positive attitudes, with 60.3% agreeing or totally agreeing, while 18.4% disagreed or totally disagreed. Similarly, for translational research, 59.6% agreed or totally agreed, with 18.2% disagreed or totally disagreed. In contrast, teaching received more neutral responses, with 30.6% selecting neutral and only 34.5% agreeing or totally agreeing, while 34.9% disagreed or totally disagreed.

Further analysis revealed that attitudes toward using animals in basic research varied significantly by gender and field of study (Table 3). Regarding gender, men expressed higher agreement (74.2%) compared to women (55.6%), while disagreement was more frequent among women (21.3%) than men (9%), and this result was statistically significant (χ^2^_(4)_ = 27.1, *p* < 0.001; V = 0.206). Similarly, differences emerged by field of study: students from science degrees showed stronger agreement (67.9%) than those from humanities degrees (43.9%), whereas disagreement was higher among humanities students (28.7%) compared to science students (13.6%), being also highly statistically significant (χ^2^_(4)_ = 41.9, *p* < 0.001; V = 0.253), indicating a moderate effect size in both cases.

Differences in support for translational research were also evident across demographic and academic factors (Table 3). When considering gender, the proportion of participants who agreed or strongly agreed was notably higher among men (74.7%) compared with women (53.2%), whereas disagreement was more common among women (22.1%) than men (8.4%). This pattern was statistically highly significant (χ^2^_(4)_ = 37.3, *p* < 0.001; V = 0.242). Field of study also showed a clear divide: students enrolled in science degrees expressed greater endorsement (66.7%) than those in humanities degrees (43.9%), while disagreement was substantially higher among humanities students (25.8%) compared to their science counterparts (14.7%). The association was significant and of moderate strength (χ^2^_(4)_ = 43.6, *p* < 0.001; V = 0.258).

Attitudes toward the use of laboratory animals for university teaching followed a similar pattern to that observed for research activities (Table 3). Participants identified as men were more likely to agree or totally agree (51.7%) compared to women (27.5%), while disagreement was higher among women (38.8%) than men (24.2%) (χ^2^_(4)_ = 33.4, *p* < 0.001; V = 0.230). Likewise, students in science degrees expressed greater agreement (39.5%) than those in humanities degrees (23.5%), whereas disagreement was more frequent among humanities students (38.6%) compared to science students (33.3%) (χ^2^_(4)_ = 32.6, *p* < 0.001; V = 0.223). No significant differences were observed based on sexual orientation, area of residence, or year of study for basic and translational research, or university teaching.

Among the personality variables, emotional stability and empathy showed only very small correlations with support for animal use and therefore were not considered meaningful. In contrast, higher levels of anthropomorphism were consistently associated with lower support for animal use across all contexts, with correlations approaching a moderate size: basic research (rho = −0.28, *p* < 0.001), translational research (rho = −0.33, *p* < 0.001), and university teaching (rho = −0.24, *p* < 0.001).

### 3.3. Participants’ Opinions on Animal Use to Solve Human Health Problems Across Species

**Figure 2:**
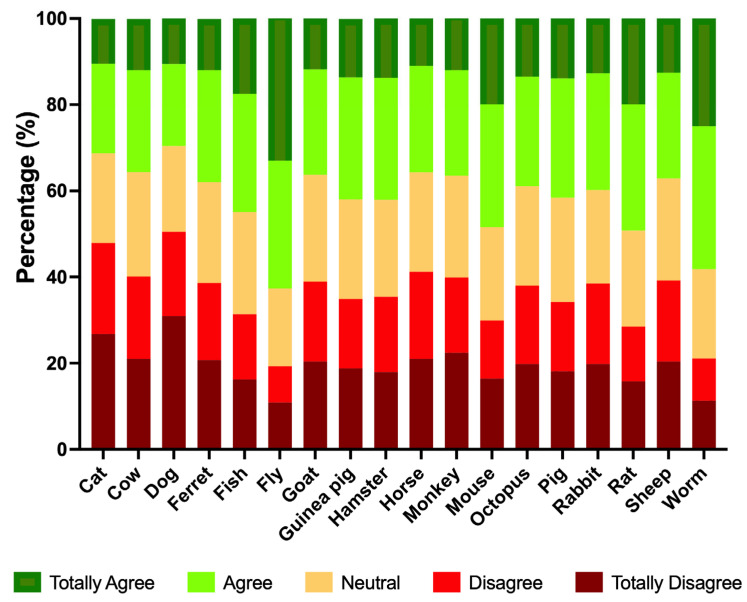
Percentage of responses to the question “Scientists should be allowed to experiment on the following animals if this can help solve human health problems,” by species. Participants were presented with a predetermined list of species and asked to rate each one from “totally disagree” to “totally agree”.

When asked whether scientists should be allowed to experiment on animals to solve human health problems, responses differed substantially by species (Figure 2; Supplementary Table S2). Companion animals attracted the strongest opposition: dogs (50.5% disagree or totally disagree vs. 29.6% agree or totally agree) and cats (47.9% vs. 31.2%). Monkeys faced relative resistance (39.9% vs. 36.6%). Livestock species tended to elicit mixed views with modest opposition and moderate support: pigs (34.2% disagree or totally disagree vs. 41.6% agree or totally agree), sheep (39.2% vs. 37.1%), goats (38.9% vs. 36.3%), cows (40.1% vs. 35.5%), and horses (41.2% vs. 35.7%). Among mammals typically used in laboratories, acceptance was comparatively higher. Rats showed the greatest support, with 28.5% disagree or totally disagree vs. 49.3% agree or totally agree, followed by mice (29.9% vs. 48.4%), hamsters (35.4% vs. 42.1%), and guinea pigs (34.9% vs. 41.9%). Rabbits were more evenly split (38.5% vs. 39.8%), and ferrets displayed a similar balance (38.6% vs. 37.9%). Finally, invertebrates and aquatic species attracted the greatest support. Flies had the strongest support, with 19.3% disagree or totally disagree vs. 62.7% agree or totally agree, followed by worms (21.1% vs. 58.2%) and fish (31.4% vs. 44.9%). Octopus elicited nearly equal levels of opposition and support (38.0% disagree or totally disagree vs. 38.9% agree or totally agree).

**Figure 3:**
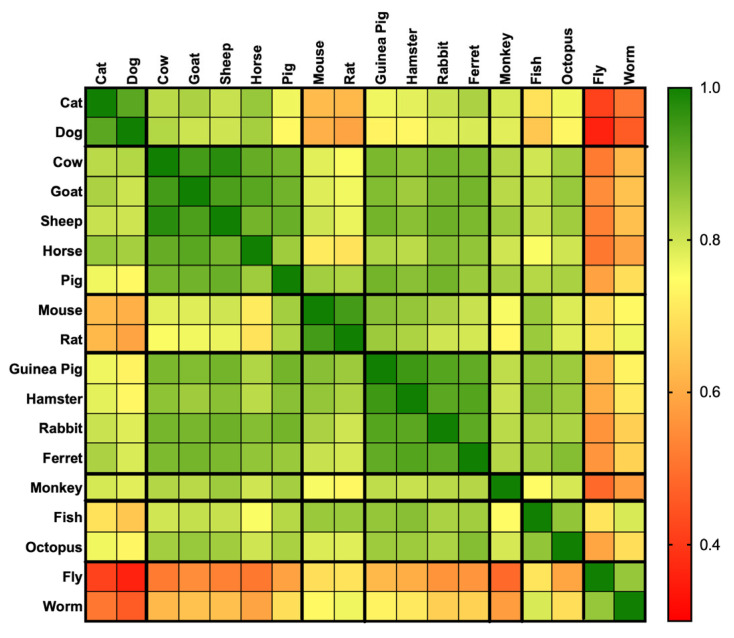
Spearman correlation matrix (rho) reordered by species categories (pets, livestock or farm animals, laboratory rodents and small mammals, monkeys, aquatics and, invertebrates). Black lines indicate group boundaries. Correlations are colour-coded from green (rho = 1, strongest correlation) to red (rho =0.2).

Support for the use of different animal species was consistently positive and strongly intercorrelated (all *p* < 0.001), although the strength of these associations varied systematically (Figure 3; Supplementary Table S3). High correlations were observed among closely related or functionally similar species, forming distinct clusters: companion animals (dog–cat rho = 0.92), livestock (cow–sheep rho = 0.98; goat–cow rho = 0.95; horse–goat rho = 0.92; pig–goat rho = 0.90), laboratory rodents (mouse–rat rho = 0.95) and small mammals (mouse–rat rho = 0.95; guinea pig–hamster rho = 0.95; rabbit–ferret rho = 0.92). Monkeys showed strong associations with both livestock and small mammals (e.g., monkey–sheep rho = 0.85; monkey–rabbit rho = 0.82), but weaker links to invertebrates (monkey–fly rho = 0.49). Octopus was most closely aligned with fish (rho = 0.86) and exhibited broad positive correlations with mammals (ferret rho = 0.88; goat rho = 0.86), while remaining less connected to insects (fly rho = 0.59). Invertebrates clustered together and with fish (worm–fly rho = 0.86; worm–fish rho = 0.79; fly–fish rho = 0.70), but showed the weakest ties to pets, with the lowest coefficients between fly and dog (rho = 0.36) and fly and cat (rho = 0.42).

## 4. Discussion

The present study showed that students that participate in our study expressed moderate support for animal use in research, but acceptance declined notably when considering its role in teaching. This pattern aligns with Spanish national trends of generally favourable views toward biomedical animal research [22,23], yet the current findings suggest a more cautious and conditional form of support marked by ethical reservations. This nuanced stance reflects international evidence that attitudes toward animal experimentation are highly context-dependent and often ambivalent rather than clearly supportive or opposed [21]. The high proportion of neutral responses, especially regarding teaching, may indicate uncertainty or reluctance to take firm positions on socially sensitive issues.

Gender patterns in this study mirror broader national trends: women showed lower agreement with the use of laboratory animals, consistent with extensive evidence that they express greater concern for animal welfare and less support for animal experimentation [21,22,23,24,39,40]. The field of study also shaped attitudes. Science students were more supportive of animal use than those in the humanities, likely due to greater exposure to scientific practice and familiarity with regulatory frameworks, both of which are known to increase acceptance [22,24,40]. Humanities students, by contrast, may rely more on ethical perspectives that emphasize harm avoidance or moral equality across species, views that align with Spain’s strong welfare sensitivity [15,28]. In this context, students without scientific training may adopt more cautious positions, whereas those with scientific backgrounds may regard animal use as ethically acceptable within regulated systems.

Anthropomorphism showed moderate negative correlations with support for animal use across contexts, consistent with previous research indicating that individuals who attribute human-like qualities to animals tend to be more critical of practices that may compromise animal welfare [41,42]. In contrast, personality traits, particularly emotional stability, and empathy showed only very small correlations with support for animal use and are therefore of limited practical significance. This indicates that, although emotionally reactive or highly empathetic students may experience some discomfort with animal use, these traits do not meaningfully influence attitudes in our sample. These findings differ from previous reports suggesting that lower emotional stability is associated with reduced support for animal use, such as in Spanish veterinary students who expressed greater concern about certain forms of animal use, particularly in research and management contexts [43].

In our study, attitudes toward animal experimentation followed a clear species-based hierarchy. At one end, companion animals (cats and dogs) elicited the strongest opposition; at the other, invertebrates (worms and flies), and to a lesser extent rodents, received the highest acceptance, with livestock and laboratory mammals occupying an intermediate position. This pattern reflects a well-documented phylogenetic moral gradient and aligns with international evidence [24,26,32,44], further supporting the view that moral status varies by species [45,46,47]. Spanish public responses illustrate this asymmetry: a recent petition against using Beagles in a toxicity study drew over a million signatures [48], whereas news of the first transplant of a genetically modified pig heart into a human prompted far less backlash [49]. The growing role of pigs in biomedical applications (e.g., chimeric organ research) likely reinforces these divergent moral framings across species [50].

Despite the strengths of the large sample size and comprehensive assessment of species-specific attitudes, several limitations must be acknowledged. First, the sample overrepresented women and students from scientific fields, which may partly limit generalizability. Second, the cross-sectional design cannot determine causal relationships between psychological traits and attitudes. Third, self-report measures may be subject to social desirability bias, particularly given the highly sensitive nature of animal research. Additionally, the large number of comparisons increases the risk of Type I error. Future studies should examine how exposure to structured ethics training, hands-on laboratory experience, or multimedia educational modules affects attitudes over time. Additionally, qualitative investigations could deepen our understanding of the moral reasoning underlying species preferences and help identify how specific narratives shape public intuitions.

## 5. Conclusions

Taken together, the demographic and psychological differences observed in this study reflect broader patterns in Spain: acceptance of animal research persists but remains conditional, species-dependent, and guided by moral intuitions. As Spain and the European Union advance toward reduced animal use and more sophisticated alternatives, understanding public expectations, such as those expressed by the student population, will be essential.

## Figures and Tables

**Table 1:** Participants’ demographic information. Number of participants and the percentage for each demografic category.

	n (%)
Gender	
Women (cis/trans)	457 (69.9%)
Men (cis/trans)	178 (27.3%)
Non binary	8 (1.2%)
Preferred not to answer	10 (1.5%)
Sexual Orientation	
Asexual	13 (7.1%)
Bisexual	150 (24.2%)
Heterosexual	419 (67.6%)
Homosexual	38 (6.1%)
Prefer not to answer	33 (5.1%)
Area of residence	
Rural	136 (20.8%)
Urban	517 (79.2%)
Field of Study	
Sciences	448 (68.6%)
Humanities	205 (31.4%)
Most Advanced Year Enrolled	
1st Year	271 (41.5%)
2nd Year	104 (15.9%)
3rd Year	104 (15.9%)
4th Year	161 (24.7%)
5th Year	7 (1.1%)
6th Year	6 (0.9%)
Total (n)	653

**Table 2:** Participants’ scores on psychological variables, including personality subscales, empathy, and anthropomorphism. Scores are presented as mean and standard deviation (SD). Higer scores indicate stronger expression of each trait.

	Mean	SD
Personality		
Agreeableness	11	2.13
Conscientiousness	10.1	2.56
Emotional stability	8.54	2.83
Extraversion	8.42	2.96
Openness	10.2	2.21
Empathy	34.4	5.03
Anthropomorphism	36.2	8.47

**Table 3:** Participants’ attitudes toward Basic Research, Translational Research, and University Teaching by gender and field of study.

	Totally Disagree	Disagree	Neutral	Agree	Totally Agree
	n (%)	n (%)	n (%)	n (%)	n (%)
Basic Research					
Women (cis/trans)	46 (10.1)	51 (11.2)	106 (23.2)	168 (36.8)	86 (18.8)
Men (cis/trans)	6 (3.4)	10 (5.6)	30 (16.9)	71 (39.9)	61 (34.3)

Humanities	29 (14.1)	30 (14.6)	56 (27.3)	65 (31.7)	25 (12.2)
Sciences	25 (5.6)	36 (8.0)	83 (18.5)	176 (39.3)	128 (28.6)
Translational Research					
Women (cis/trans)	46 (10.1)	55 (12)	113 (24.7)	168 (36.8)	75 (16.4)
Men (cis/trans)	8 (4.5)	7 (3.9)	30 (16.9)	71 (39.9)	62 (34.8)

Humanities	29 (14.1)	24 (11.7)	62 (30.2)	71 (34.6)	19 (9.3)
Sciences	27 (6)	39 (8.7)	83 (18.5)	174 (38.8)	125 (27.9)
University Teaching					
Women (cis/trans)	84 (18.4)	93 (20.4)	154 (33.7)	82 (17.9)	44 (9.6)
Men (cis/trans)	21 (11.8)	22 (12.4)	43 (24.2)	58 (32.6)	34 (19.1)

Humanities	52 (25.4)	27 (13.2)	78 (38)	28 (13.7)	20 (9.8)
Sciences	59 (13.2)	90 (20.1)	122 (27.2)	115 (25.7)	62 (13.8)

## Data Availability

The raw data supporting the findings of this study are included in Supplementary Table S4. Further datasets generated during the study will be made available by the corresponding author upon reasonable request.

## Supplementary Materials

The following supporting information can be downloaded at https://www.mdpi.com/article/10.3390/ani16071005/s1: Table S1: Participants’ attitudes toward animal use in Basic Research, Translational Research, and University Teaching.; Table S2: Participants’ responses to the question “Scientists should be allowed to experiment on the following animals if this can help solve human health problems,” by species; and Table S3: Pearson correlation by species. *** *p* < 0.001. Table S4. Raw data from the study.

## Abbreviations

The following abbreviations are used in this manuscript:

3R	3Rs: replacement, reduction, and refinement
CEISH	Ethics Committee for Human-Related Research
EMA	European Medicines Agency
EU	European Union
FDA	Food and Drug Administration
NCad	Netherlands National Committee
SD	Standard Deviation
US	United States
