# Supplementary material for: How University Students Evaluate the Use of Laboratory Animals: The Role of Species and Individual Differences
Source: Animals (Basel). 2026 Mar 25;16(7):1005. doi: 10.3390/ani16071005 (PMC13072222; doi:10.3390/ani16071005)
Supplement: Supplementary file 1 [file animals-16-01005-s001.zip › Supplementary Table S1.pdf]

Supplementary Table S1. Participants' attitudes toward animal use in Basic Research, Translational Research, and University Teaching.

	Basic Research		Translational Research		University Teaching	
	n	%	n	%	n	%
Totally Disagree	54	8.3	56	8.6	111	17.0
Disagree	66	10.1	63	9.6	117	17.9
Neutral	139	21.3	145	22.2	200	30.6
Agree	241	36.9	245	37.5	143	21.9
Totally Agree	153	23.4	144	22.1	82	12.6
