# Supplementary material for: How University Students Evaluate the Use of Laboratory Animals: The Role of Species and Individual Differences
Source: Animals (Basel). 2026 Mar 25;16(7):1005. doi: 10.3390/ani16071005 (PMC13072222; doi:10.3390/ani16071005)
Supplement: Supplementary file 1 [file animals-16-01005-s001.zip › Supplementary Table S2.pdf]

Supplementary Table S2. Participants' responses to the question "Scientists should be allowed to experiment on the following animals if this can help solve human health problems," by species.

	**Cat**		**Cow**		**Dog**		**Ferret**		**Fish**		**Fly**		**Goat**		**Guinea pig**		**Hamster**		**Horse**		**Monkey**		**Mouse**		**Octopus**		**Pig**		**Rabbit**		**Rat**		**Sheep**		**Worm**	
	n	%	n	%	n	%	n	%	n	%	n	%	n	%	n	%	n	%	n	%	n	%	n	%	n	%	n	%	n	%	n	%	n	%	n	%
**Totally Disagree**	175	26.8	137	21.0	202	30.9	135	20.7	106	16.2	71	10.9	133	20.4	123	18.8	117	17.9	137	21.0	146	22.4	107	16.4	129	19.8	118	18.1	129	19.8	103	15.8	133	20.4	74	11.3
**Disagree**	138	21.1	125	19.1	128	19.6	117	17.9	99	15.2	55	8.4	121	18.5	105	16.1	114	17.5	132	20.2	114	17.5	88	13.5	119	18.2	105	16.1	122	18.7	83	12.7	123	18.8	64	9.8
**Neutral**	136	20.8	159	24.3	130	19.9	153	23.4	155	23.7	118	18.1	162	24.8	151	23.1	147	22.5	151	23.1	154	23.6	142	21.7	151	23.1	158	24.2	142	21.7	145	22.2	155	23.7	135	20.7
**Agree**	136	20.8	154	23.6	124	19.0	170	26.0	179	27.4	193	29.6	160	24.5	185	28.3	185	28.3	161	24.7	160	24.5	186	28.5	166	25.4	181	27.7	177	27.1	192	29.4	160	24.5	217	33.2
**Totally Agree**	68	10.4	78	11.9	69	10.6	78	11.9	114	17.5	216	33.1	77	11.8	89	13.6	90	13.8	72	11.0	79	12.1	130	19.9	88	13.5	91	13.9	83	12.7	130	19.9	82	12.6	163	25.0
