# Supplementary material for: How University Students Evaluate the Use of Laboratory Animals: The Role of Species and Individual Differences
Source: Animals (Basel). 2026 Mar 25;16(7):1005. doi: 10.3390/ani16071005 (PMC13072222; doi:10.3390/ani16071005)
Supplement: Supplementary file 1 [file animals-16-01005-s001.zip › Supplementary Table S3.pdf]

Supplementary Table S3. Pearson correlation by species. *** $p < 0.001$.

	**Cat**	**Cow**	**Dog**	**Ferret**	**Fish**	**Fly**	**Goat**	**Guinea Pig**	**Hamster**	**Horse**	**Monkey**	**Mouse**	**Octopus**	**Pig**	**Rabbit**	**Rat**	**Sheep**	**Worm**
**Cat**	—	0.821***	0.922***	0.837***	0.696***	0.416***	0.837***	0.766***	0.776***	0.859***	0.793***	0.631***	0.765***	0.765***	0.806***	0.624***	0.807***	0.509***
**Cow**	0.821***	—	0.829***	0.885***	0.799***	0.516***	0.946***	0.888***	0.868***	0.910***	0.829***	0.780***	0.846***	0.893***	0.894***	0.754***	0.975***	0.625***
**Dog**	0.922***	0.829***	—	0.789***	0.650***	0.359***	0.802***	0.727***	0.738***	0.843***	0.780***	0.609***	0.733***	0.739***	0.784***	0.588***	0.801***	0.462***
**Ferret**	0.837***	0.885***	0.789***	—	0.847***	0.563***	0.894***	0.914***	0.929***	0.863***	0.827***	0.807***	0.877***	0.856***	0.917***	0.795***	0.887***	0.669***
**Fish**	0.696***	0.799***	0.650***	0.847***	—	0.703***	0.810***	0.861***	0.875***	0.753***	0.744***	0.856***	0.863***	0.826***	0.838***	0.854***	0.808***	0.790***
**Fly**	0.416***	0.516***	0.359***	0.563***	0.703***	—	0.547***	0.625***	0.606***	0.511***	0.486***	0.693***	0.591***	0.586***	0.560***	0.700***	0.528***	0.859***
**Goat**	0.837***	0.946***	0.802***	0.894***	0.810***	0.547***	—	0.882***	0.851***	0.924***	0.823***	0.783***	0.857***	0.898***	0.892***	0.763***	0.938***	0.642***
**Guinea Pig**	0.766***	0.888***	0.727***	0.914***	0.861***	0.625***	0.882***	—	0.953***	0.832***	0.816***	0.874***	0.851***	0.897***	0.927***	0.854***	0.896***	0.727***
**Hamster**	0.776***	0.868***	0.738***	0.929***	0.875***	0.606***	0.851***	0.953***	—	0.821***	0.807***	0.861***	0.851***	0.872***	0.922***	0.836***	0.873***	0.711***
**Horse**	0.859***	0.910***	0.843***	0.863***	0.753***	0.511***	0.924***	0.832***	0.821***	—	0.800***	0.714***	0.800***	0.850***	0.877***	0.699***	0.896***	0.588***
**Monkey**	0.793***	0.829***	0.780***	0.827***	0.744***	0.486***	0.823***	0.816***	0.807***	0.800***	—	0.756***	0.792***	0.844***	0.822***	0.736***	0.851***	0.577***
**Mouse**	0.631***	0.780***	0.609***	0.807***	0.856***	0.693***	0.783***	0.874***	0.861***	0.714***	0.756***	—	0.786***	0.846***	0.837***	0.946***	0.800***	0.741***
**Octopus**	0.765***	0.846***	0.733***	0.877***	0.863***	0.591***	0.857***	0.851***	0.851***	0.800***	0.792***	0.786***	—	0.840***	0.836***	0.782***	0.849***	0.692***
**Pig**	0.765***	0.893***	0.739***	0.856***	0.826***	0.586***	0.898***	0.897***	0.872***	0.850***	0.844***	0.846***	0.840***	—	0.897***	0.833***	0.907***	0.692***
**Rabbit**	0.806***	0.894***	0.784***	0.917***	0.838***	0.560***	0.892***	0.927***	0.922***	0.877***	0.822***	0.837***	0.836***	0.897***	—	0.799***	0.902***	0.664***
**Rat**	0.624***	0.754***	0.588***	0.795***	0.854***	0.700***	0.763***	0.854***	0.836***	0.699***	0.736***	0.946***	0.782***	0.833***	0.799***	—	0.770***	0.766***
**Sheep**	0.807***	0.975***	0.801***	0.887***	0.808***	0.528***	0.938***	0.896***	0.873***	0.896***	0.851***	0.800***	0.849***	0.907***	0.902***	0.770***	—	0.638***
**Worm**	0.509***	0.625***	0.462***	0.669***	0.790***	0.859***	0.642***	0.727***	0.711***	0.588***	0.577***	0.741***	0.692***	0.692***	0.664***	0.766***	0.638***	—
